# An ancestry informative marker panel design for individual ancestry estimation of Hispanic population using whole exome sequencing data

**DOI:** 10.1186/s12864-019-6333-6

**Published:** 2019-12-30

**Authors:** Li-Ju Wang, Catherine W. Zhang, Sophia C. Su, Hung-I H. Chen, Yu-Chiao Chiu, Zhao Lai, Hakim Bouamar, Amelie G. Ramirez, Francisco G. Cigarroa, Lu-Zhe Sun, Yidong Chen

**Affiliations:** 1 Greehey Children’s Cancer Research Institute, University of Texas Health San Antonio, San Antonio, TX 78229 USA; 2 Department of Molecular Medicine, University of Texas Health San Antonio, San Antonio, TX 78229 USA; 3 Department of Cell Systems and Anatomy, University of Texas Health San Antonio, San Antonio, TX 78229 USA; 4 Department of Surgery, University of Texas Health San Antonio, San Antonio, TX 78229 USA; 5 Department of Population Health Sciences, University of Texas Health San Antonio, San Antonio, TX 78229 USA; 6 Institute for Health Promotion Research, University of Texas Health San Antonio, San Antonio, TX 78229 USA

**Keywords:** Admixture, Ancestry Informative Markers (AIMs), Hispanics population, STRUCTURE, Whole exome sequencing, Hepatocellular carcinoma

## Abstract

**Background:**

Europeans and American Indians were major genetic ancestry of Hispanics in the U.S. These ancestral groups have markedly different incidence rates and outcomes in many types of cancers. Therefore, the genetic admixture may cause biased genetic association study with cancer susceptibility variants specifically in Hispanics. For example, the incidence rate of liver cancer has been shown with substantial disparity between Hispanic, Asian and non-Hispanic white populations. Currently, ancestry informative marker (AIM) panels have been widely utilized with up to a few hundred ancestry-informative single nucleotide polymorphisms (SNPs) to infer ancestry admixture. Notably, current available AIMs are predominantly located in intron and intergenic regions, while the whole exome sequencing (WES) protocols commonly used in translational research and clinical practice do not cover these markers. Thus, it remains challenging to accurately determine a patient’s admixture proportion without additional DNA testing.

**Results:**

In this study we designed an unique AIM panel that infers 3-way genetic admixture from three distinct and selective continental populations (African (AFR), European (EUR), and East Asian (EAS)) within evolutionarily conserved exonic regions. Initially, about 1 million exonic SNPs from selective three populations in the 1000 Genomes Project were trimmed by their linkage disequilibrium (LD), restricted to biallelic variants, and finally we optimized to an AIM panel with 250 SNP markers, or the UT-AIM250 panel, using their ancestral informativeness statistics. Comparing to published AIM panels, UT-AIM250 performed better accuracy when we tested with three ancestral populations (accuracy: 0.995 ± 0.012 for AFR, 0.997 ± 0.007 for EUR, and 0.994 ± 0.012 for EAS). We further demonstrated the performance of the UT-AIM250 panel to admixed American (AMR) samples of the 1000 Genomes Project and obtained similar results (AFR, 0.085 ± 0.098; EUR, 0.665 ± 0.182; and EAS, 0.250 ± 0.205) to previously published AIM panels (Phillips-AIM34: AFR, 0.096 ± 0.127, EUR, 0.575 ± 0.290, and EAS, 0.330 ± 0.315; Wei-AIM278: AFR, 0.070 ± 0.096, EUR, 0.537 ± 0.267, and EAS, 0.393 ± 0.300). Subsequently, we applied the UT-AIM250 panel to a clinical dataset of 26 self-reported Hispanic patients in South Texas with hepatocellular carcinoma (HCC). We estimated the admixture proportions using WES data of adjacent non-cancer liver tissues (AFR, 0.065 ± 0.043; EUR, 0.594 ± 0.150; and EAS, 0.341 ± 0.160). Similar admixture proportions were identified from corresponding tumor tissues. In addition, we estimated admixture proportions of The Cancer Genome Atlas (TCGA) collection of hepatocellular carcinoma (TCGA-LIHC) samples (376 patients) using the UT-AIM250 panel. The panel obtained consistent admixture proportions from tumor and matched normal tissues, identified 3 possible incorrectly reported race/ethnicity, and/or provided race/ethnicity determination if necessary.

**Conclusions:**

Here we demonstrated the feasibility of using evolutionarily conserved exonic regions to infer admixture proportions and provided a robust and reliable control for sample collection or patient stratification for genetic analysis. R implementation of UT-AIM250 is available at https://github.com/chenlabgccri/UT-AIM250.

## Background

Over the past several hundred years, the America continent has been the hot spot attracting people from different continental populations that were originally separated by geography, such as African (mass migration due to Atlantic slave trade), European (the age of exploration and Spanish colonization of the Americas), and Asian (California gold rush) [1]. Due to meeting and mixing of previously isolated populations through the years, the resulting *population admixture* carries novel genotypes with new genetic variations inherited from a variety of ancestral populations [2]. In other words, admixed individuals have a genetic mosaic of ancestry that distinguishes them from their parental populations.

Hispanics in the U.S. have genetic ancestry from European, African and Native American. The admixture population presents opportunity for the study of health disparity due to disease susceptibility [3, 4] or drug response [5–7]. In cancer study, it has been shown Hispanics have clearly different cancer incidence rates and outcomes [8]. The pattern of genetics and DNA variations of Hispanic individuals was affected by many historical events [9]. Therefore, genetic admixture may bias estimates of associations with cancer susceptibility genes in Hispanics. The investigation of population structure and admixture proportion is also important in disease diagnosis. For example, the incidence rate of liver cancer has been shown to be very different between Hispanic/Asian and non-Hispanic white populations [10], especially the Hispanic population in South Texas [11, 12]. To estimate the admixture proportion of individuals, most published ancestry informative marker (AIM) panels were designed using up to a few hundred genome-wide ancestry-informative single nucleotide polymorphisms (SNPs) that exhibit large variation in minor allele frequency (MAF) among populations that are usually located in non-exonic regions [13–16]. To estimate the admixture proportion, several model-based clustering approaches have been developed for the determination of the genetic ancestry of human and other organisms. Pritchard et al. used a Bayesian algorithm STRUCTURE to first define the populations and then assign individuals to them [17]. An efficiently implemented algorithm, ADMIXTURE, incorporated a similar Bayes inference model, which enabled the analysis of AIM panels with thousands of markers [18]. More algorithms for estimating genetic ancestry can be found in the literature [19].

Recently, whole exome sequencing (WES) has become a standard protocol in translational research and clinical diagnostics to identify the underlying genetic cause of diseases due to the fact that most pathogenic variants are located in exonic regions and the drastically reduced cost of WES [20–22]. WES provides detailed information of genetic variants including rare genetic events and unknown somatic mutations between different genetic conditions for large cohort of patients. Particularly in translational research, WES offers an unbiased view than conventional targeted molecular diagnostics approach, commonly available in many large genomic studies such as The Cancer Genome Atlas (TCGA) [23]. Previous studies showed that admixture proportions could be determined by using principal component analysis (PCA) with all variants [24], using allele frequency for pooled DNA [25], and using off-target sequence reads [26]. However, a panel of AIM within exome, if feasible, will allow rapid determination of a patient’s ancestry admixture from WES data and thus validate self-reported race/ethnicity.

In this study, we aimed to re-tune an AIM design pipeline to precisely determine ancestry admixture of Hispanic populations using WES data. Using the 1000 Genomes Project data, we selected SNPs that have different MAF of African (AFR), European (EUR), and East Asian (EAS) populations and quantified by *I*_*n*_-statistics. We validated our optimal panel with 250 AIMs using the admixed American (AMR) of the 1000 Genomes Project, and compared our results to several published AIM panels with SNPs designed mostly in intronic/intergenic regions. Finally, we applied our AIM panel to TCGA-LIHC data and an in-house hepatocellular carcinoma (HCC) study with self-reported Hispanic patients enrolled in South Texas.

## Methods

### Population samples

We use the 1000 Genomes Phase III Whole Genome Sequencing (WGS) data as the resource to identify AIMs [27]. Data was downloaded for each chromosome, excluding Mitochondrial, chrX, and chrY (ftp://ftp.1000genomes.ebi.ac.uk/vol1/ftp/). The 1000 Genomes Phase III data were aligned with hg19 human reference genome. The SNPs were then extracted by ancestral populations (Table 1) using VCFtools [28] and BCFtools [29]. Individuals from the Caribbean and African Americans were excluded from the ancestral population of Africa due to high levels of admixture observed. The Vietnamese population was also excluded from the East Asian ancestral population. Additionally, in order to eliminate Hispanics white interference, we pruned the Iberian population in Spain from the European population. For validation purpose, we utilized the entire admixed American (AMR) collection, including Mexican Ancestry from LA, Puerto Ricans, Colombians and Peruvians (Table 1) to validate our panel.

**Table 1:** Populations of the 1000 Genomes Project included in this study

Super population	Subpopulation	# of samples
East Asian (EAS)	Chinese Dai in Xishuangbanna (CDX), Han Chinese (CHB), Southern Han Chinese (CHS), Japanese in Tokyo, Japan (JPT)	405
African (AFR)	Esan in Nigeria (ESN), Gambian in Western Division, the Gambia (GWD), Luhya in Webuye, Kenya (LWK), Mende in Sierra Leone (MSL), Yoruba in Ibadan, Nigeria (YRI)	504
European (EUR)	Utah residents (CEPH) with European Ancestry (CEU), Finnish in Finland (FIN), British in England and Scotland (GBR), Toscani in Italia (TSI)	396
Admixed American (AMR)	Colombian in Medellin, Colombia (CLM), Mexican Ancestry in Los Angeles, California (MXL), Peruvian in Lima, Peru (PEL), Puerto Rican in Puerto Rico (PUR)	347

The populations were downloaded from the 1000 Genomes Project database. We excluded Vietnamese from EAS, African American from AFR, and Iberian of Spain from EUR (see Methods)

### Data processing and AIMs generation

The genome-wide data from the 1000 Genomes Project were first constrained to exonic region. Obtained SNPs were further subject to linkage disequilibrium filtering (*r*^2^ < 0.2, plink option: --r2), allele frequency (AF) calculation, and minor allele frequency (MAF < 0.01, plink option: --maf 0.01) elimination by PLINK (using vcftools to convert all three ancestral populations to .ped format with option --plink). The output files from PLINK were processed by the AIM generator (python script, AIMs_generator.py) [30]. This python script, provided by Daya *et. al*, performs LD pruning and select AIMs based on Rosenberg’s *I*_*n*_ Statistic [31] which defines the informativeness of SNPs,

1 $$ {I}_n=-\left({p}_A\ln \left({p}_A\right)+{p}_a\ln \left({p}_a\right)\right)+\left(\frac{1}{K}\sum \limits_{i=1}^K{p}_{i,A}\ln \left({p}_{i,A}\right)+\frac{1}{K}\sum \limits_{i=1}^K{p}_{i,a}\ln \left({p}_{i,a}\right)\right), $$

where *p*_*A*_ and *p*_*a*_ are the frequencies of 2 alleles across all individuals for a given marker, and *p*_*i,A*_ and *p*_*i,a*_ are the corresponding allele frequencies in the *i*^th^ population. If a marker is unique in the *i*^th^ population only, the second term in Eq. (1) will be 0, or *I*_*n*_ will be the largest, while *I*_*n*_ = 0 if the marker is equally distributed among all populations. To design our AIM panel, we first obtained nested subsets of AIMs up to 5000 candidate SNPs (see Additional file 1**:** Table S1; python code AIMs_generator.py, with ldfile/bim files from PLINK, ldthresh = 0.1, distances = 100,000, strategy = *I*_*n*_). We expected 5000 SNP candidates would allow us to select robust AIM panel considering SNPs with balanced *I*_*n*_ from overall population, as well as least bias between pair-wise *I*_*n*_. The ancestry distribution of AIMs was provided in Table 2.

**Table 2:** Proportions of AIMs among three ancestral populations

# of AIMs	African	East Asian	European
10	4 (40%)	2 (20%)	4 (40%)
50	20 (40%)	12 (24%)	18 (36%)
100	40 (40%)	28 (28%)	32 (32%)
250	90 (36%)	80 (32%)	80 (32%)
500	172 (34%)	165 (33%)	163 (33%)
750	256 (34%)	265 (35%)	229 (31%)
1000	329 (33%)	355 (36%)	316 (32%)
2000	616 (31%)	763 (38%)	621 (31%)
3000	920 (31%)	1124 (38%)	956 (32%)
4000	1251 (31%)	1488 (37%)	1261 (32%)
5000	1582 (32%)	1810 (36%)	1608 (32%)

AIMs are determined by AIM_generator.py script. We examined AF of each population for each AIM to assign the SNP to the dominant population (presented as the number of SNPs and percentage in each AIM panels). Note that larger AIM panels are not necessary contain markers in smaller panels due to the requirement of balancing number of markers in 3 populations

### Optimal AIM panel selection

Ancestral proportions were inferenced by STRUCTURE [17] and ADMIXTURE [18]. The error of estimation was determined by the results of STRUCTURE and ADMIXTURE:

2 $$ {e}_k=1/{N}_k{\sum}_{i\in \left\{{k}^{th} population\right\}}\left(1.0-{f}_{k,i}\right), $$

where we assume *f*_*k,i*_ is the admixture proportion of *i*^th^ person’s identified *k*^th^ population (ideally 100% in *k*^th^ population), and *k* = {EUR, EAS, and AFR}. A person will be classified into *k*^th^ population if he/she has a maximum *k*^th^ population proportion estimated by STRUCTURE and ADMIXTURE, thus we can estimate the error according to Eq. (2).

The optimal number of AIMs were determined when the observed accuracy, (1− *e*_*k*_), of classified known population did not improve by adding more candidate SNPs within the 5000-SNP pool. We selected AIMs with an optimal balance in three populations (Table 2) from pair-wise *I*_*n*_ statistics. The final 250 AIMs (UT-AIM250) and its *I*_*n*_ Statistics were provided in Additional file 2: Table S2.

### WES of HCC samples

WES was performed with Illumina HiSeq 3000 system at the GCCRI Genome Sequencing Facility, using Illumina’s TruSeq Rapid Exome Library Prep kit (Illumina, CA) which covers ~ 45 Mb with 99.45% of NCBI RefSeq regions. All exomeCapture sequencing was performed with 100 bp paired-end (PE) module, and pooled 6 samples per lane with targeted ~100x fold coverage. Paired reads were aligned to human reference genome hg19 (the same genome build used by the 1000 Genomes Project) with Burrows-Wheeler Aligner (BWA) [32]. Duplicated reads were removed by SAMtools [33] and Picard (http://broadinstitute.github.io/picard) and realigned with GATK [34] considering dbSNPs information. Variants were identified by VarScan [35]. To report any variant statistics on locations specified by AIMs, we only required a minimum coverage of 2 and no variant calling threshold.

### PCA of AIM genotypes

PCA was performed on dataset of multi-locus genotypes to identify population distribution of each individual. The genotype matrix was obtained by applying the “read.vcfR” function of the R package [36]. Then, we converted the genotype to numeric numbers (0|0 = 0, 1|0 or 0|1 = 1, 1|1 = 2, and .|. = NA) by the Admixture_gt2PCAformat function (see the github site). For PCA, we utilized dudi.pca (from “ade4” R package [37]). If there were missing values, we used estim_ncpPCA (“missMDA” R package [38]) to fill NA in genotype matrix before performing PCA.

### Performance evaluation of AIM panel

To assess the robustness of AIM panel that separates 3 continental populations, we first projected three populations into 3D space using PCA as described previously. We assume each population follows multi-variate normal distribution,

$$ {f}_k\left(x;{\mu}_k,{\Sigma}_k\right)=\frac{1}{\sqrt{\left(\mid {\Sigma}_k\mid {\left(2\pi \right)}^d\right)}}\exp \left(-\frac{1}{2}\left(x-{\mu}_k\right){\Sigma}_k^{-1}{\left(x-{\mu}_k\right)}^{\hbox{'}}\right), $$

where *μ*_*k*_ is 1x*d* mean vector (here *d* = 3) of the *k*^th^ population, and Σ_*k*_ is a *d*-by-*d* co-variance matrix. After estimation of the multivariate distributions of all 3 continental populations, we estimated the probability of mis-classified samples from one population to the other two when the probability of a given sample with known population origin was lower than those assigned to the other two groups, or the misclassification probability of samples in *i*^th^ population into *j*^th^ population is ${P}_m\left(i,j\right)={\iint}_{\left\{x:{f}_i(x)<{f}_j(x)\right\}}{f}_i\left(x;{\mu}_i,{\Sigma}_i\right)$. We report the overall mis-classification probability, *P*_*AIM*_ = ∑_*all i* ≠ *j*_*P*_*m*_(*i*, *j*) as a measure of the capacity separating populations using a specific AIM panel. A smaller *P*_AIM_ indicates less chance of a sample to be misclassified using a given AIM panel, or in other words, farther separation between 3 populations.

### SNP processing of HCC patients

We started by pruning in-house WES data from 26 HCC patients with matched adjacent non-tumor (Adj. NT) and tumor. Initial pruning was performed by sequencing depth of each SNP, and only biallelic SNPs were considered (vcftools options: --min-alleles 2 --max-alleles 2 --recode). A SNP was eliminated if it had more than 10% missing genotype across all samples by VCFtools (vcftools options: --max-missing 0.9 --recode).

### SNP processing of TCGA–LIHC samples

We extracted specific SNP positions of UT-AIM250 from 788 TCGA-LIHC samples (376 patients) by using GDC BAM slicing tool (https://docs.gdc.cancer.gov/API/Users_Guide/BAM_Slicing/). The tool enables to download specific regions of BAM files instead of the whole BAM file for a given TCGA sample. These BAM slices were then processed with VarScan to determine variant fraction as described in previous sub-sections. The TCGA-LIHC whole exome data were derived from 4 sample types **(**Fig. 5a**)**. According to race and ethnicity in clinical data of TCGA-LIHC, we re-classified 7 population groups (White, Asian, Black, Hispanic White, Reported as Hispanic, American Indian or Alaska Native, and Unknown) **(**Fig. 5a**)**. The SNPs were selected if it has more than 90% genotype throughout all sample by VCFtools, and further required biallelic SNPs.

## Results

### AIMs panel design and admixture estimation pipeline

**Fig. 1:**
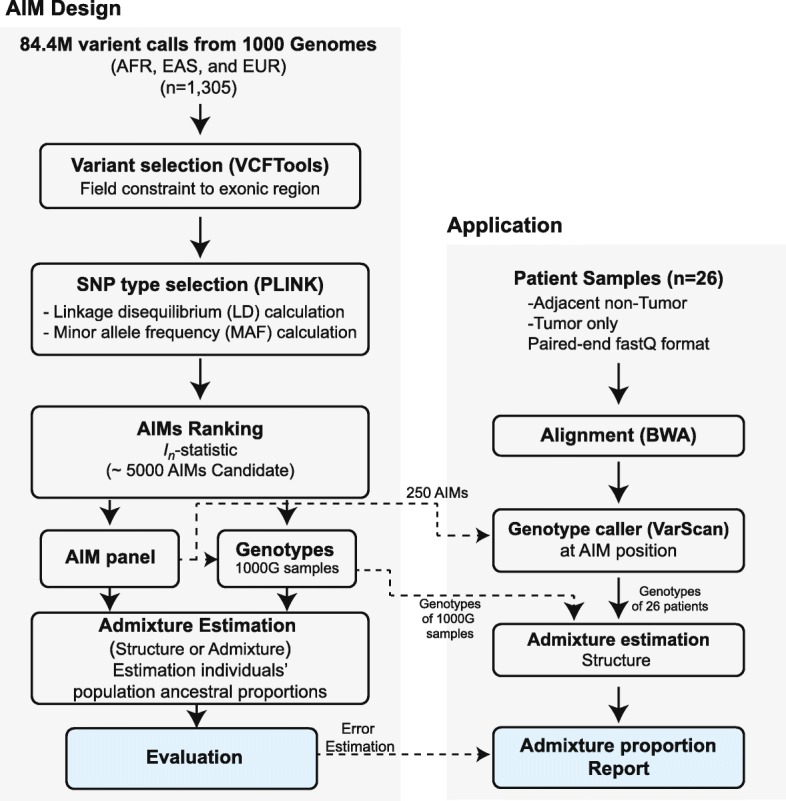
Flowchart of our AIM panel design and analysis pipeline. The pipeline is separated into two parts, AIM panel design (AIM Design) and Ancestral proportion estimation application (Application). For the AIM Design pipeline (left panel), variant files from the 1000 Genomes Project (*n* = 1305) were position filtered to exonic region by VCFTools. The variant files were calculated linkage disequilibrium (LD) and minor allele frequency (MAF) by PLINK. SNPs were selected as AIMs based on *I*_*n*_-statistic for overall population or each continental population. Finally, population ancestral proportions were estimated by STRUCTURE. For the Application pipeline (right panel), the 26 HCC tumors with matched Adj. NT data were processed by standard WES analysis pipeline using BWA, GATK and genotype caller VarScan at AIM positions. The last step in this panel was admixture estimation and reported the ancestral proportions of individual

We aim to design an AIM panel for estimating admixture proportions for the Hispanic population using WES data. We first focused our selection of continental population from the 1000 Genomes Project, removing all possible sources of biases (removing African American from AFR collection and Iberian of Spain from EUR collection, and Vietnamese which are further down south of Asia; see Methods). We then constrained the ancestral markers within the exome. Figure 1 outlined the flowchart of our AIM panel design pipeline (left panel). Here we assumed that our targeted population was comprised of three ancestry components: African (AFR), East Asian (EAS), and European (EUR). For this study, we focused only on SNPs (about 84.8 million variants in total) that were extracted from three ancestry populations (*n* = 1305) in the 1000 Genomes Project (Table 1). These SNPs were then filtered based on positions to ~ 1 million exonic SNPs using VCFTools. To confirm these markers are good AIM candidate SNPs, all SNPs were pruned by following criteria: (1) linkage disequilibrium (LD) *r*^2^ < 0.2 within 100 kb window to avoid redundancy, (2) minor allele frequency (MAF) < 0.01 to avoid sequencing artifact, and (3) evaluation of ancestral informativeness by using Eq. (1) *I*_*n*_-statistic for all pair-wise comparisons of 3 continental populations as described in the Methods section. A total of 100,295 SNPs met the first 2 criteria, and among them, we generated AIMs panels with 10, 50, 100, 250, 500, and up to 5000 AIMs (see Table 2, and Additional file 1: Table S1).

### Comparisons of population structure tools and selection of optimal AIM panel

**Fig. 2:**
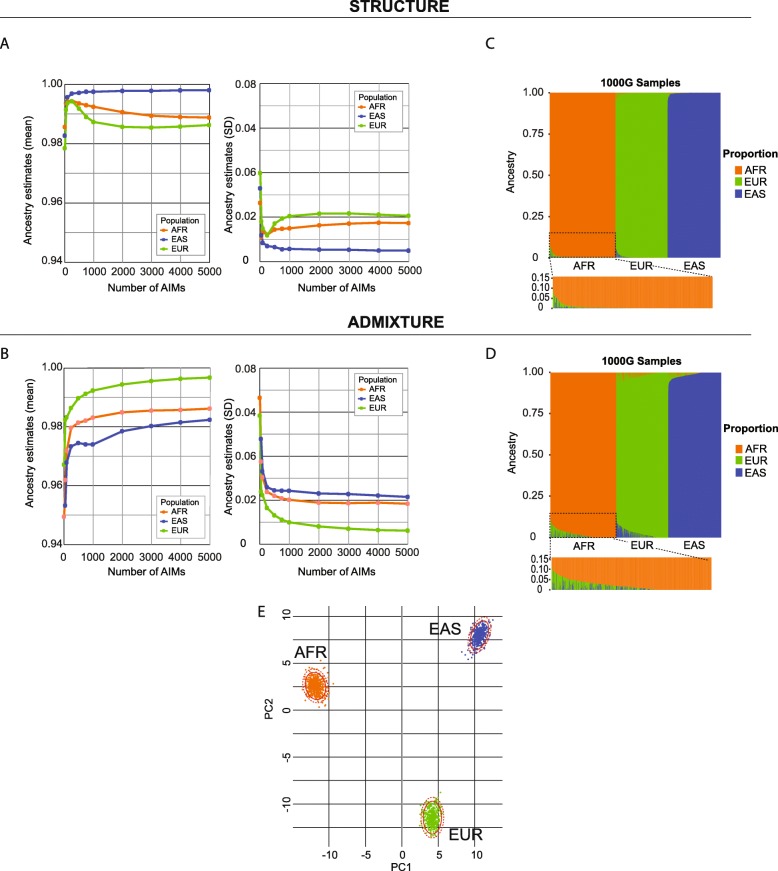
Selection of a tool for ancestral population proportion estimation. The results were presented as those from STRUCTURE (**a**, **c**) and from ADMIXTURE (**b**, **d**). **(a**, **b)** Performance of AIM panels with different number of markers. Mean and SD were plotted for each population. At 250 markers, the accuracy plateaus when STRUCTURE algorithm is used. **(c**, **d)** Proportion plot for ancestral populations on 250 AIMs using STRUCTURE and ADMIXTURE. The populations were ordered by groups: AFR: African, EUR: European, and EAS: East Asian. Individuals in (**d**) were ordered identically to (**c**). **(e)** PCA plots for three ancestral populations on 250 AIMs

Here we compared the two popular admixture tools, STRUCTURE and ADMIXTURE. These two tools utilized different algorithms (Bayesian statistics vs maximum likelihood estimation) to estimate population structure. The efficiency of ADMIXTURE is known to be higher with multi-thread capability compared to STRUCTURE without much compromise in accuracy. As expected, the accuracy of STRUCTURE in population estimation was better than ADMIXTURE (both set at *K* = 3) (Fig. 2a, b). For each population and its corresponding ancestral proportion estimation, the mean and standard deviation (SD) of ancestry estimation accuracy of STRUCTURE and ADMIXTURE were AFR: 0.991 ± 0.016 vs 0.977 ± 0.027 (one-tailed *t*-test *P* = 7.20 × 10^− 23^), EUR: 0.988 ± 0.021 vs 0.969 ± 0.034 (*P* = 1.70 × 10^− 20^), and EAS: 0.996 ± 0.009 vs 0.989 ± 0.017 (*P* = 2.92 × 10^− 13^). With 250 AIMs, we observed the best grouping accuracy and lowest SD in three ancestral populations with the STRUCTURE algorithm (AFR: 0.995 ± 0.012, EUR: 0.994 ± 0.012, and EAS: 0.997 ± 0.007), while ADMIXTURE required more than 250 AIMs to gain desirable accuracy (Fig. 2a, b). Examining individual estimations carefully from both algorithms further confirmed that ADMIXTURE was less robust (Fig. 2c, d; much longer green tail in Fig. 2d, inset for the AFR population). For these reasons, subsequent analysis was focused on the 250-AIM panel (termed as UT-AIM250 thereafter) and the STRUCTURE algorithm for admixture proportion estimation. Within the UT-AIM250 panel, we identified 90 African AIMs (36%), 80 European AIMs (32%), and 80 East Asian AIMs (32%) (see Table 2 and Additional file 2: Table S2). The ranges of *I*_n_ for pair-wise ancestral populations were: AFR vs EUR: (0 to 0.614), AFR vs EAS: (1.185 × 10^− 5^ to 0.623); and EAS vs EUR: (0 to 0.645), and overall population (0.134 to 0.569) (Additional file 2: Table S2). We utilized genotypes from three ancestry populations (*n* = 1305) in the 1000 Genomes Project on UT-AIM250 panel and confirmed that the UT-AIM250 panel had sufficient discriminating capacity to separate three ancestral populations (Fig. 2e, with 95% and 99% confidence ranges denoted by solid and dash circles, respectively).

### Comparisons between the UT-AIM250 panel and published 34-AIM and 278-AIM panels

**Fig. 3:**
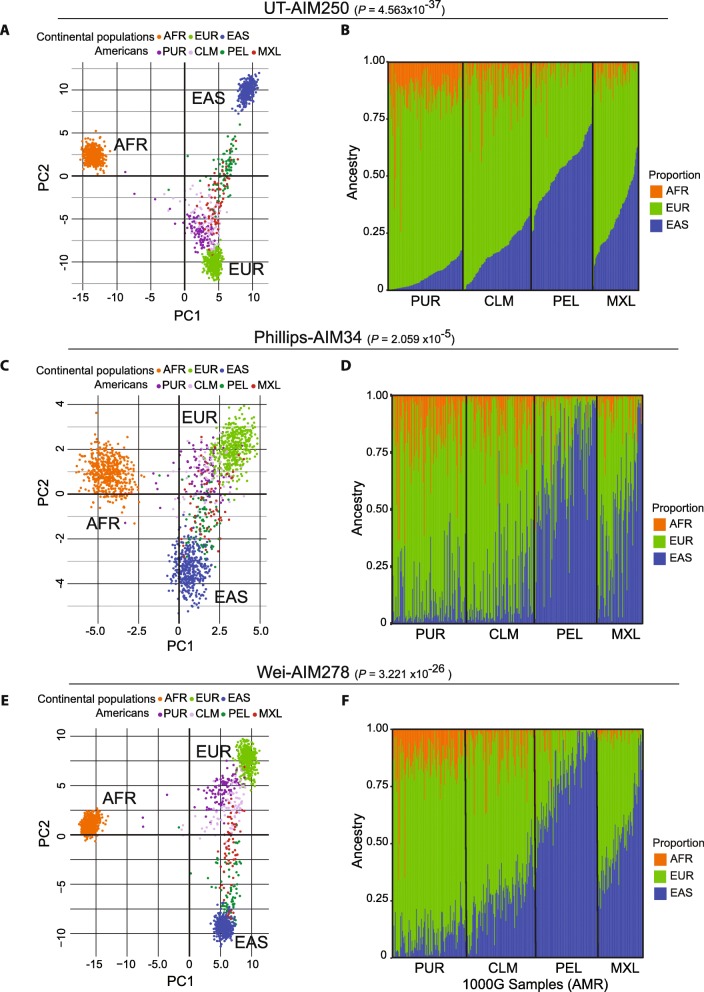
Comparisons between the proposed UT-AIM250 panel and two published AIM panels. (**a**, **c**, **e**) PCA plots for AMR population distributions on UT-AIM250, Phillips-AIM34, and Wei-AIM278 panels. **(b, d, f)** Proportion plots for admixed Americans (AMR). Individuals are ordered within each population group. PUR: Puerto Rican; CLM: Colombian; PEL: Peruvian and MXL: Mexican in LA

We compared our UT-AIM250 panel and two published panels, 34 AIM-panel [14] (Phillips-AIM34) and 278 AIM-panel [39] (Wei-AIM278), on the Admixed American (AMR) population of the 1000 Genomes Project. These panels were originally generated from the three continental populations (AFR, EUR, and EAS) with slightly different inclusion criterion and samples available at the time. The Phillips-AIM34 panel is composed of SNPs in both exonic regions (2 SNPs) and non-exonic regions (32 SNPs); the Wei-AIM278 panel is composed of SNPs in exonic (3 SNPs) and non-exonic regions (275 SNPs). Figure 3 depicts the results from UT-AIM250 (Fig. 3a, b), Phillips-AIM34 (Fig. 3c, d) and Wei-AIM278 panels (Fig. 3e, f) of 3 continental ancestral populations plus Admixed American (AMR). The AMR was composed of four subpopulations, Colombian (CLM), Mexican in LA (MXL), Peruvian (PEL), and Puerto Rican (PUR). Following the analysis pipeline (Fig. 1, right panel), genotypes of the AIMs of the three panels were extracted from AMR (*n* = 347) and 3 continental populations (*n* = 1305). The admixture of populations was estimated by STRUCTURE and plotted by both bar charts and principal component plots (Fig. 3). All three panels can separate continental populations, and UT-AIM250 achieved a much superior separation (Fig. 3a, c, e), with misclassification probability *P*_UT-AIM250_, *P*_Phillips-AIM34_, and *P*_Wei-AIM278_ of 4.563 × 10^− 37^, 2.059 × 10^− 5^, and 3.221 × 10^− 26^, respectively (see the Methods section). The population structure showed a very similar trend among the three panels (Fig. 3b, d, f): within AMR sub-populations, Puerto Rican had much higher European ancestral proportions (AFR: 0.149 ± 0.109, EUR: 0.789 ± 0.111, and EAS: 0.062 ± 0.051), while Peruvian had strong influence from East Asian (AFR: 0.032 ± 0.066, EUR: 0.449 ± 0.111 and EAS: 0.519 ± 0.124), in line with previous published studies [13, 40, 41]. For MXL, the proportions of 3 ancestral populations were AFR = 0.046 ± 0.046, EUR = 0.634 ± 0.142, and EAS = 0.320 ± 0.149. Pearson correlation confirmed an overall agreement among the three panels (Table 3; 0.70, 0.83 and 0.85 between UT-AIM250 and Phillips-AIM34; 0.89, 0.93 and 0.96 between UT-AIM250 and Wei-AIM278 for AFR, EUR and EAS ancestral proportions, respectively). Similar correlation coefficients for each sub-population can be found in Table 3.

**Table 3:** Pearson correlation coefficients between UT-AIM250 and published panels of the AMR population

Panel	Population	PUR (*n* = 104)	CLM (*n* = 94)	PEL (*n* = 85)	MXL (*n* = 64)	All (*n* = 347)
Phillips-AIM34	AFR-AFR ρ	0.67 (*P* = 5.97 × 10^− 15^)	0.57 (*P* = 2.11 × 10^− 9^)	0.83 (*P* = 1.46 × 10^− 22^)	0.22 (*P* = 7.56 × 10^− 2^)	0.70 (*P* = 5.83 × 10^− 52^)
	EUR-EUR ρ	0.69 (*P* = 9.72 × 10^− 16^)	0.67 (*P* = 9.39 × 10^− 14^)	0.57 (*P* = 1.60 × 10^− 8^)	0.81 (*P* = 4.09 × 10^− 16^)	0.83 (*P* = 7.30 × 10^− 91^)
	EAS-EAS ρ	0.26 (*P* = 6.87 × 10^− 3^)	0.42 (*P* = 2.00 × 10^− 5^)	0.64 (*P* = 4.18 × 10^− 11^)	0.77 (*P* = 1.49 × 10^− 13^)	0.85 (*P* = 4.75 × 10^− 96^)
Wei-AIM278	AFR-AFR ρ	0.89 (*P* = 1.96 × 10^− 37^)	0.86 (*P* = 1.75 × 10^− 28^)	0.89 (*P* = 2.29 × 10^− 30^)	0.40 (*P* = 9.75 × 10^− 4^)	0.89 (*P* = 1.20 × 10^− 122^)
	EUR-EUR ρ	0.80 (*P* = 1.74 × 10^− 24^)	0.84 (*P* = 1.55 × 10^− 26^)	0.83 (*P* = 6.24 × 10^− 23^)	0.92 (*P* = 1.24 × 10^− 26^)	0.93 (*P* = 1.60 × 10^− 152^)
	EAS-EAS ρ	0.47 (*P* = 3.89 × 10^− 7^)	0.73 (*P* = 7.51 × 10^− 17^)	0.89 (*P* = 1.01 × 10^− 29^)	0.93 (*P* = 8.98 × 10^− 29^)	0.96 (*P* = 6.04 × 10^− 193^)

Pearson correlation coefficient (*p*-value)

### Ancestry estimation for HCC patients

**Fig. 4:**
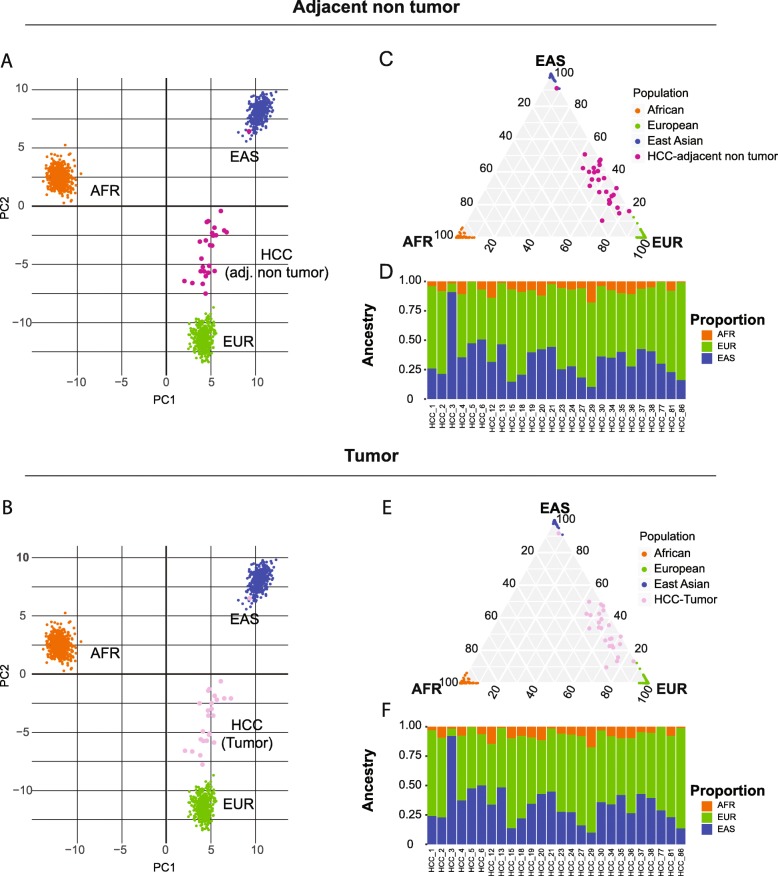
Application to 26 HCC tumors with matched adjacent non-tumor using WES data**. (a, b)** PCA plots from HCC adjacent non-tumor samples and HCC tumor samples. **(c, e)** Triangle plots for ancestral division probability of HCCs from African (AFR), East Asian (EAS), and European (EUR). **(d, f)** Proportion plots for HCCs, ordered by patient ID

The key to design UT-AIM250 is to validate self-reported race/ethnicity of Hispanic patients for translational study without adding specific ancestral markers to standard exome capture kits for sequencing library preparation. We applied the UT-AIM250 panel to estimate the ancestral proportion of a collection of 26 HCC patients (all self-reported as Hispanic from San Antonio or South Texas regions) with matched tumor tissues and Adj. NT tissues. We extracted genotypes of 250 SNPs from Adj. NT and tumors using VarScan (see Methods), merged with 1305 continental populations from the 1000 Genomes Project, and visualized using the first 2 principal components (Fig. 4a for Adj. NT and b for tumor only). No obvious differences were observed between Adj. NT and tumor samples, indicating the feasibility of using tumor data alone to assess the patient ancestral proportion. We calculated ancestral components by STRUCTURE (*K* = 3). The ancestral proportions of our HCC patients are AFR = 0.065 ± 0.044, EUR = 0.595 ± 0.151, and EAS = 0.340 ± 0.163, similar to those of MXL. In triangle plots (Fig. 4c, e**)**, HCC patients were mostly aligned along the axis of EAS and EUR, similar to the PCA plot. One patient (HCC-3) was predicted as Asian (in the Asian population in PCA plot, and Asian proportion = 0.916; Fig. 4d, f), so we excluded this patient from subsequent genetic analysis. Similar to the comparison between STRUCTURE and ADMIXTURE algorithm, we examined the correlation coefficient ρ between tumor tissues and Adj. NT tissues. The results were 0.96, 0.99 and 0.99 for AFR-AFR, EUR-EUR, and EAS-EAS, respectively (all *P* < 10^− 14^). Taken together, our UT-AIM250 panel is accurate and robust to determine the ancestral proportion from normal or even tumor samples.

### Ancestry estimation for TCGA-LIHC samples

**Fig. 5:**
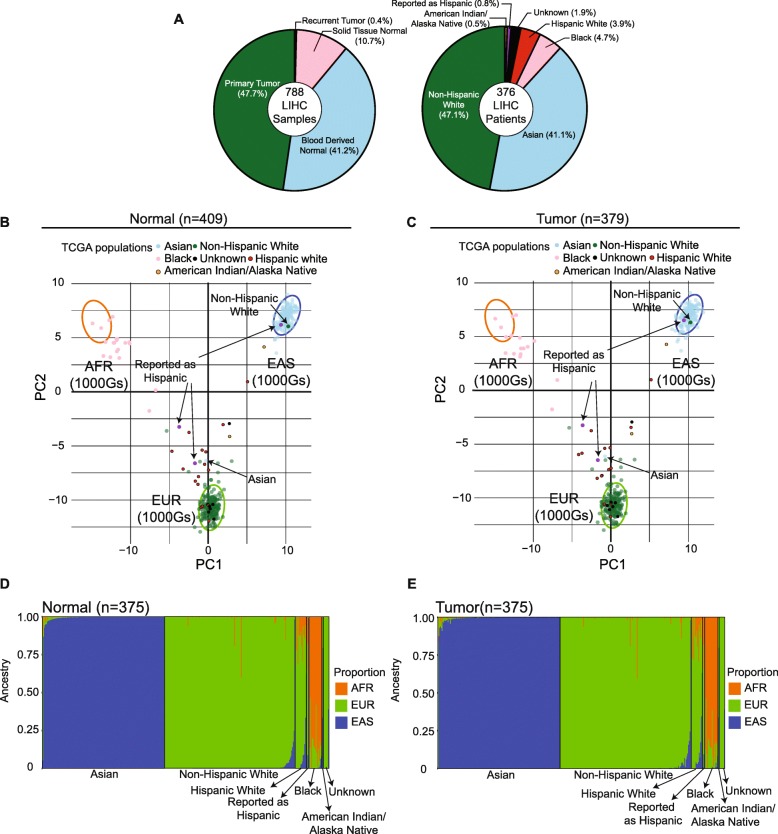
Application to 788 TCGA-LIHC samples (376 patients). **(a)** Summary of TCGA-LIHC samples and patients. Left and right pie charts are the break-down of sample types and self-reported race and ethnicity of all LIHC patients. **(b, c)** PCA plots of 788 TCGA-LIHC samples (normal: *n* = 409; tumor: *n* = 379). Normal group includes DNAs derived from blood and/or solid tissue normal, and tumor group includes primary tumor and recurrent tumor. Purple points were these from patients “Reported as Hispanic”. The confidence interval depicted by three ellipses (determined from 3 continental population EAS, EUR and AFR of the 1000 Genomes Project) is 0.99. **(d, e)** Proportion plots for 375 TCGA-LIHC patients with matched normal (blood derived normal: *n* = 325; solid tissue normal: *n* = 50) and primary tumor samples, ordered by sample ID

In order to verify the accuracy of UT-AIM250 on different samples, we evaluated all TCGA-LIHC 376 patients and compared to their self-reported race/ethnicity. TCGA-LIHC has a total of 788 samples with WES data, derived from 4 sample types (41.2% blood derived normal, 10.7% solid tissue normal, 47.7% primary tumor, and 0.4% recurrent tumor, Fig. 5a left panel, and Additional file 3: Table S3**)**. Based on race and ethnicity of each patient reported, we divided all 376 patients to 7 populations (47.1% White, 41.1% Asian, 4.7% Black, 3.9% Hispanic white, 0.8% Reported as Hispanic, 0.5% American Indian or Alaska Native, and 1.9% unknown, Fig. 5a right panel, and Additional file 3: Table S3). We applied UT-AIM250 to all 788 samples (normal *n* = 409, and tumor *n* = 379). The PCA plots showed similar patterns in both normal and tumor (Fig. 5b for normal and c for tumor only), indicating our UT-AIM250 panel is robust even if normal DNA is not available. In Fig. 5b-e, we selected 375 TCGA-LIHC patients with matched primary tumor and normal samples (325 blood derived normal and 50 solid tissue normal), excluding TCGA-BC-4072 which had primary tumor sample only. We utilized STRUCTURE (*K* = 3) to calculate ancestral components (Additional file 3: Table S3). The ancestral proportions of 375 TCGA-LIHC patients were plotted with bar chart (Fig. 5d for normal, e for primary tumor). Two patients, TCGA-DD-AACA and TCGA-ZS-A9CF, had three sample types, blood derived normal, primary tumor, and recurrent tumor. We compared the ancestral proportions of three sample types on each patient, and the results were consistent (TCGA-DD-AAC: EAS = 0.999, EUR = 0.001, and AFR = 0; TCGA-ZS-A9CF: EAS = 0.001, EUR = 0.999, and AFR = 0.001). Our analysis also concluded that there were three patients (TCGA-G3-A5SI, TCGA-G3-AAUZ, and TCGA-FV-A4ZQ) with mismatched race/ethnicity from their self-reported data. TCGA-G3-A5SI (self-reported as Asian) was predicted as white (EUR proportion = 0.826; Fig. 5b-c). We also predicted both patients TCGA-G3-AAUZ (self-reported as Hispanics) and TCGA-FV-A4ZQ (self-reported as White) to be Asian (EAS proportion = 0.992, and 0.984, respectively). In addition, 7 patients with unknown race/ethnicity status were assigned to their corresponding genetic groups. Therefore, the SNP positions of our UT-AIM25 is unaffected by possible tumor mutations and UT-AIM250 is a robust panel of ancestral markers within exome.

## Discussion

In this study, we developed, validated and tested the pipeline for designing AIM panels within the evolutionarily conserved exome regions to distinguish genetic ancestry descendants base on three continental populations (African, European, and East Asian). Although WES could be applied to analyze population structure using all variants [24], it may be problematic since variants will be influenced by the number of somatic mutations in tumor samples, which typically are significantly different on germline and tumor [42–44]. By using UT-AIM250 panel and we acquired satisfactory performance by removing low-frequency MAFs and applying constraints with only biallelic SNPs even with the tumor samples. To further reduce the impact of somatic mutations on our AIM panel design, one may choose to filter SNPs using COSMIC database [45] or other relevant tumor variant collections, such as the International Cancer Genome Consortium (ICGC) [46]. While the number of our HCC patients is small, we believe it is sufficient to demonstrate the utility of WES data to identify ancestry proportion of individuals. In some clinical applications in which only tumor samples are available, our UT-AIM250 is proved to be a cost-effective tool to confirm the race and ethnicity of patients when WES data are available.

The AIMs were selected from three continental populations (African, European, and East Asian). These populations were the major groups which contributed to the ancestral genetic variety of people in the U.S. through various migration routes [47]. There are variable phenotypes of Hispanics in the U.S. [48], and it is recognized that health disparity does exist in different populations, even within Hispanic populations [49] due to their diverse genetic background such as populations shown in Fig. 3a (AMR subpopulations). We have carefully selected subpopulations specifically for our targeted population, such as removing of Iberian (IBS), to further constrain EUR to be considered as Non-Hispanic White (NHW).

Future studies may use the Native American as one of the continental populations. However, as shown in Fig. 3a, ancestral components of AMR subpopulations of the 1000 Genomes Project are quite diverse. We will continue evaluating other genomic resources, preferably WGS, to include richer genetic information from the Native American that are commonly accepted as an ancestral population. Asian was chosen in this study not only due to its stable genome variation, but also because of the convincing evidence that one of the origin ancestries of Native American could be Asian who came from northeast Asia by passing Beringia strait [50, 51]. We believe our AIM panel is sufficient to identify distinct genetic groups for downstream data analysis, such as risk factor assessment.

Along with the development of precision medicine, the population determination plays an important role [52]. Both in our HCC patients or TCGA-LIHC patients, we observed the problem about accuracy of patients’ self-reported race/ethnicity status. After ancestral estimation, the results of some patients do not match what were reported. Due to several potential factors, such as native language, environment, immigration, etc., patients sometimes mis-report their real race/ethnicity, especially in an immigrant society. Thus, UT-AIM250 could correct this mistake and provide reliable ancestral report if WES data are availablle.

There are many different types of variants besides SNPs, such as insertions, deletions, and haplotypes. In this study, we focused on biallelic SNPs only. Extending to insertions and deletions may complicate the analysis due to the precise definition of these variants in each patient. Recognized that, in the population genetic field, these potential factors are typically considered and analyzed on the distribution of population proportions [53], future studies may extend our work to incorporate more types of variants into the AIM panel design.

## Conclusions

Here we constructed a unique AIM panel, UT-AIM250, designed within the evolutionarily conserved exonic regions, to determine the admixture proportions of three continental populations (AFR, EUR, and EAS) for Hispanic in South Texas. We demonstrated the accuracy using AMR subpopulations from the 1000 Genomes Project and compared to the published Phillips-AIM34 and Wei-AIM278 panels. We further applied our panel to 26 Hispanic HCC patients and 375 TCGA-LIHC patients with matched tumor and adjacent non-tumor tissues. The estimated ancestral proportions showed no significant difference between non-tumor and tumor tissues, enabling us to evaluate patients’ tumor specimens to verify self-reported Hispanic patients and/or their specific genetic analysis groups. Since WES is one of the dominant genome-wide variant analysis platforms, the UT-AIM250 panel offers a cost-effective yet accurate method for the determination of patients’ ancestral composition. R implementation of UT-AIM250 is available at https://github.com/chenlabgccri/UT-AIM250.

## Supplementary information

**Additional file 1: Table S1.** Informativeness of AIMs across different panels.

**Additional file 2: Table S2.** Informativeness of AIMs of the UT-AIM250 panel.

**Additional file 3: Table S3.** The ancestral proportions and clinical data of TCGA-LIHC samples (S3A: tumor DNAs; S3B: normal DNAs).

## Data Availability

R implementation and example of UT-AIM250 is available at https://github.com/chenlabgccri/UT-AIM250.

## Abbreviations

Adj. NT: Adjacent non-tumor
AFR: African
AIM: Ancestry informative marker
AMR: Admixed American
BWA: Burrows-Wheeler Aligner
CLM: Colombian
EAS: East Asian
EUR: European
HCC: Hepatocellular carcinoma
LD: Linkage disequilibrium
MAF: Minor allele frequency
MXL: Mexican in LA
PCA: Principal component analysis
PE: Paired-end
PEL: Peruvian
PUR: Puerto Rican
SD: Standard deviation
SNPs: Single nucleotide polymorphisms
TCGA: The Cancer Genome Atlas
TCGA-LIHC: TCGA Liver Hepatocellular Carcinoma
WES: Whole exome sequencing
WGS: Whole genome sequencing
